# An Open Platform for Seamless Sensor Support in Healthcare for the Internet of Things

**DOI:** 10.3390/s16122089

**Published:** 2016-12-08

**Authors:** Jorge Miranda, Jorge Cabral, Stefan Rahr Wagner, Christian Fischer Pedersen, Blaise Ravelo, Mukhtiar Memon, Morten Mathiesen

**Affiliations:** 1 Centro Algoritmi, University of Minho, Guimarães 4800-058, Portugal; jcabral@dei.uminho.pt; 2 Section of Electrical and Computer Engineering, Department of Engineering, Aarhus University, Aarhus N 8200, Denmark; sw@eng.au.dk (S.R.W.), cfp@eng.au.dk (C.F.P.); 3 Normandy University UNIROUEN, ESIGELEC, IRSEEM, Rouen F-76000, France; blaise.ravelo@esigelec.fr; 4 Information Technology Center, Sindh Agriculture University, Tandojam 70060, Pakistan; mukhtiar.memon@sau.edu.pk; 5 Sekoia, Aarhus 8000, Denmark; morten@sekoia.dk

**Keywords:** e-Health, pervasive healthcare, telemedicine, ambient assisted living, telecare, sensors, internet of things, health devices, open source, IEEE 11073

## Abstract

Population aging and increasing pressure on health systems are two issues that demand solutions. Involving and empowering citizens as active managers of their health represents a desirable shift from the current culture mainly focused on treatment of disease, to one also focused on continuous health management and well-being. Current developments in technological areas such as the Internet of Things (IoT), lead to new technological solutions that can aid this shift in the healthcare sector. This study presents the design, development, implementation and evaluation of a platform called Common Recognition and Identification Platform (CRIP), a part of the CareStore project, which aims at supporting caregivers and citizens to manage health routines in a seamless way. Specifically, the CRIP offers sensor-based support for seamless identification of users and health devices. A set of initial requirements was defined with a focus on usability limitations and current sensor technologies. The CRIP was designed and implemented using several technologies that enable seamless integration and interaction of sensors and people, namely Near Field Communication and fingerprint biometrics for identification and authentication, Bluetooth for communication with health devices and web services for wider integration with other platforms. Two CRIP prototypes were implemented and evaluated in laboratory during a period of eight months. The evaluations consisted of identifying users and devices, as well as seamlessly configure and acquire vital data from the last. Also, the entire Carestore platform was deployed in a nursing home where its usability was evaluated with caregivers. The evaluations helped assess that seamless identification of users and seamless configuration and communication with health devices is feasible and can help enable the IoT on healthcare applications. Therefore, the CRIP and similar platforms could be transformed into a valuable enabling technology for secure and reliable IoT deployments on the healthcare sector.

## 1. Introduction

During recent years, concerns about the efficiency and quality of public health systems has increased. In Europe, two of the factors are the population growth and aging [1,2,3] and the lack of qualified health professionals [2]. A population growth of 4% by 2050 is expected, due to factors such as dynamics of fertility, increasing migration rates and increase of life expectancy. With these demographic changes happening, the number of healthcare professionals should increase, but a shortfall of one million healthcare workers and an imbalance in their geographical distribution is projected by 2020 [2]. The first is due to undersupply by professional educational institutions and current staff aging, i.e., undersupply in rural areas and oversupply in urban areas, as more and more of these professionals move to countries where better working and professional prospects, as well as financial incentives, are offered.

With these changes happening, public health systems are starting to show their limitations: growing costs, increased numbers of medical errors and lack of adequate medical support, due to lack of human resources and time dedicated to patients’ care. These factors are starting a shift in health culture from a mortality and morbidity one, to a culture where health and well-being are encouraged [4,5]. This shift is challenging because it implies changes in several aspects [6] such as: (1) a change from reactive medicine to a proactive and preventive one; (2) decentralized treatment from hospitals to home and care institutions; and (3) empowering citizens and a change in healthcare culture to enable a collaborative medicine where the patient and medical staff are both responsible for health management.

Despite the reluctance by a part of the medical community to embrace new technologies, mainly due to suspicion about the accuracy and effectiveness of using these tools in well-defined processes [7], users tend to embrace and approve the use of technologies for health monitoring. There has been research focused on monitoring and controlling medical conditions such as high blood pressure [8,9,10], diabetes [11,12], weight loss [13,14] and Chronic Obstructive Pulmonary Disease (COPD) [15,16,17]. Using new Information and Communication Technologies (ICT) in healthcare scenarios, such as wearable sensors, mobile devices and cloud computing, provides an opportunity for continuously monitoring health behaviors, which allows building a story around a person. The necessity to study how these changes can happen has led to the emergence of newer disciplines in the ICT area such as Telecare, Telemedicine, e-Health, Ambient Assisted Living (AAL) and Pervasive Healthcare [6]. Each of them appeared on different moments of technological development, starting with the integration of the telephone for remote medical appointments and monitoring, up to the integration of large ICT systems that allow continuous monitoring. Also, the birth of mobile computing and emergence of wearable technologies have made collecting personal data more accessible. All of these possibilities led researchers to focus on how ICT can be applied in health care scenarios such as hospitals, nursing homes and patients’ homes, on an attempt to spread the responsibilities for health care between the diverse agents.

A new paradigm that focuses on pushing forward integration of technology on daily routines is the Internet of Things (IoT). Xu et al. [18] present the IoT as “*a dynamic global network infrastructure with **self-configuring capabilities** based on **standard and interoperable communication protocols** where physical and virtual ‘Things’ have identities, physical attributes, and virtual personalities and use intelligent interfaces, and are **seamlessly integrated into the information network***”. Continuous research on how the IoT can impact the development of healthcare applications is an ongoing process [19,20,21]. It is commonly agreed that the availability of wireless technologies, such as Bluetooth/Bluetooth Low Energy (BLE), IEEE 802.15.4, IEEE 802.11 (Wi-Fi), Long-Term Evolution (LTE) and 5G, and the convergence towards IP based protocols are some of the reasons for an easier integration of computational devices, such mobile devices and wireless sensor networks (WSNs) [19,22,23,24,25]. Other technologies that can also enhance integration are Radio Frequency Identification (RFID), its branch Near Field Communication (NFC), and BLE proximity sensing profiles, that allow an easier identification of “things”, either people or devices, in IoT scenarios [26]. Still, there are some challenges: the quality of service, integration and unification of different network topologies and standards at the network level; the safety and privacy of users and their data; integration of different software and hardware providers; and application of the technology itself in healthcare organizations [21,22].

With these challenges in mind, the authors have developed the CareStore platform [27], with the purpose of seamlessly deploying healthcare and AAL devices and applications in the home of citizens in need of care. As part of the platform, a gateway device called the Common Recognition and Identification Platform (CRIP) was developed to aid caregivers and citizens manage their health in a seamless way, namely by identifying users and health devices and providing easy device interaction.

The aim of this study is to present the design, development, implementation and evaluation of the CRIP. The main contributions of this work are: (1) a demonstration of the feasibility of this type of platform concerning the integration and unification different technologies used in healthcare; (2) an overview of the technical challenges found while developing and evaluating the CRIP prototypes; and (3) a presentation of guidelines for future healthcare IoT projects.

### 1.1. Related Work

Work on gateways for healthcare is quite broad, usually focusing on presenting solutions for specific healthcare deployment scenarios such as hospitals or homes. Here we highlight some related projects where some sort of gateway was developed. A summary of the technologies used by each one and where it can be applied is presented in Table 1. The field of ambient-assisted living and related healthcare services and applications and its integration on the IoT have been previously reviewed by Islam et al. [21], Memon et al. [28] and Rashvand et al. [29].

Rahmani et al. [30] presented a conceptual gateway, the Smart e-Health Gateway, aimed at healthcare applications for hospitals and private homes, and also developed a proof-of-concept gateway, the UT-GATE. The gateway comprises several communication technologies such as Bluetooth/BLE, Wi-Fi and 6LoWPAN, and it is based on two computational platforms. As a case study, the authors collected ECG data from wireless sensors, stored the data locally and also synchronized it with a remote service. The overall work focused more on the implementation of the gateway and on technical aspects that can improve its performance, reliability and, to some extent, its interoperability.

Catarinucci et al. [31] presented an IoT-aware smart architecture for automatic monitoring and tracking of patients, personnel and devices in medical facilities. As proof-of-concept, the authors implemented a Smart Hospital System, comprised of an 802.15.4/6LoWPAN WSN, with Ultra High Frequency (UHF) RFID capabilities, and mobile devices, able to collect in real time both environmental conditions and patients’ physiological parameters. The core of the system is an IoT gateway, responsible for collecting and processing data from the sensors and storing it both locally and remotely.

Cubo et al. [32] proposed a platform to overcome the challenges of appropriately managing the inter-connection of heterogeneous “things” in the ambient intelligence domain. The aim of the system is to assist patients and health providers, either in a hospital or at home, by seamlessly monitoring and detecting emergency situations. A part of the system is a wireless gateway whose main function is to retrieve and transform data collected from diverse IEEE 802.15.4-based local sensors and send it to a remote server to be stored.

Saponara et al. [33] presented a Remote Healthcare Model and Embedded Sensing/ Communication platform to support multiple chronic illnesses. The authors developed and tested the platform with three healthcare scenarios in mind: self-measurement by the patient (1:1), measurement made by a professional caregiver (1:N) and measurement by the patient on a specific healthcare facility (point-of-care). The platform is flexible enough to be adapted and used at the patients’ home or in a healthcare facility such as a pharmacy or a nursing home. The system is comprised of Bluetooth biomedical devices (custom and commercial ones), available according to one of the described deployment scenarios; an e-Health remote centre responsible for managing users and their data; and a local gateway. The latter is responsible for collecting vital data from biomedical devices, sending it to the e-Health centre, as well as performing local processing of data to provide statistics and early warnings about possible medical conditions.

Yang et al. [34] presented an open-platform-based intelligent medicine box, the iMedBox, as well as two dedicated medical sensors/devices. The box has enhanced connectivity and inter-changeability for the integration of devices and services, provided by Wi-Fi, ZigBee, RFID and a tablet. The medicine box is able to directly communicate with remote healthcare information systems, in order to configure itself according to the needs of the patient using it, as well to store its information to be accessed by medical personnel such as doctors, nurses or caregivers.

The approach of Ghose et al. [35] is based on mobile devices, i.e., they suggested using a smartphone running a healthcare application that is connected with a backend via web services. The communication with local medical sensors is made using either Bluetooth or Wi-Fi. The main contribution of this work is the mobility introduced on this type of platforms where the gateway is either represented by a PC/laptop or other dedicated device. The authors highlight this as an important aspect of their approach, especially when this type of system can be used at home, in small clinics, temporary health camps, or developing countries like India with extended rural areas and unreliable electrical power supply.

### 1.2. The CareStore Platform

**Figure 1:**
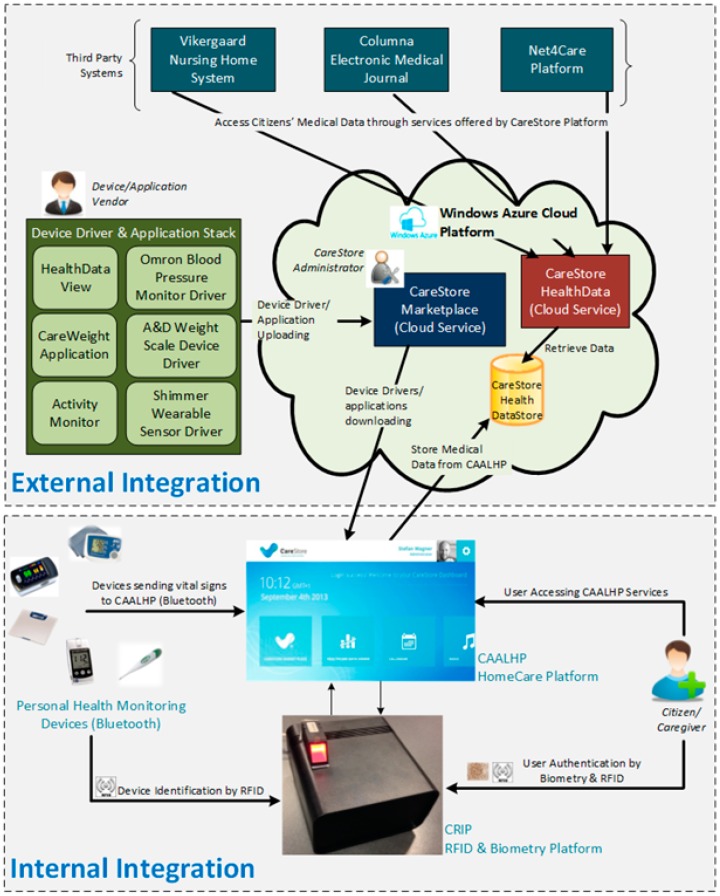
Overview of the CareStore Platform, comprised by three main systems: the CAALHP, the CRIP and the Marketplace. The first two are deployed together on a same space, such as a user’s private home, enabling the integration of local devices and users interaction with the platform. The third allows vendors to upload device drivers and profiles that can be later downloaded and installed on the CAALHP and on the CRIP.

The overall aim of the CareStore project is to develop an inexpensive and user-friendly open-source platform for supporting staff and residents’ easy deployment of devices and applications in residential apartments, without requiring technical staff intervention. The platform also allows all vendors free and easy access to publish new health devices and applications to be later installed by the users in a seamless way. As depicted in Figure 1, the platform consists of three major components:

- *CAALHP*: The Common Ambient Assisted Living Home Platform is an open-source runtime execution platform for execution of services, applications, device drivers and a user interface (UI) for staff and residents interaction with the platform;
- *CRIP*: Common Recognition and Identification Platform used to identify staff, residents and devices, using RFID and/or biometrics as identification technologies;
- *CareStore Marketplace*: An online shared platform for uploading and storing healthcare and AAL drivers and applications, that can be downloaded and installed in a seamless way on the CAALHP and the CRIP.

These three components allow the involvement of all major actors from the healthcare system in one common platform, i.e., citizens and caregivers can use the CRIP as an ambient-assisted platform for health management via applications and seamless integration with personal health devices; doctors can access citizens’ health information via integration of external medical services with the CareStore platform; and finally, support for heterogeneous personal health devices can be provided by manufactures that can deploy device drivers to the Marketplace. All this integration is based on technologies such as RFID/NFC, used for identification of people and devices, biometrics for identification and authentication of citizens and caregivers, Bluetooth for personal area networks of devices and web services for communication and integration between the platform components and external services. The integration of these technologies make the project meet the IoT, where different “things” such as devices, services and people are seamlessly connected.

Descriptions of some parts of the CareStore project were already published. Two electromagnetic characterizations were performed to verify the CRIP compliance with the standards for health devices to be deployed to hospitals and and homes. First, an electromagnetic compability (EMC) evaluation according to the EN55022 Class B standard [36], and second, an electromagnetic immunity (EMI) according to the EN 61000-4-3 standard [37]. In both cases, the CRIP complied with the requirements. Also, an evaluation of the entire CareStore platform was performed on a nursing home, involving the nurses and care staff [38]. The results regarding its use and understanding of the platform were positive, which indicates that our approach to these platforms may be a viable solution for healthcare providers.

## 2. Materials and Methods

In this section we start by presenting the requirements of the CRIP (Section 2.1), next the design of the system (Section 2.2 and Section 2.3), followed by the set of evaluation protocols that were designed in order to test its functionalities (Section 2.4).

### 2.1. Requirements

The following set of initial usability requirements for CRIP was defined:

- *Integration*: The CRIP subsystem shall provide a seamless integration with internal and external subsystems. A standard interface for easy access by other subsystems shall be provided. The major integration is made with CAALHP, which has the ability to communicate with the CareStore Marketplace in order to download device drivers/profiles and applications;
- *Device Installation and Configuration*: The device installation and configuration shall be absolutely seamless and shall involve minimal interaction with the end-user. A CareStore user must initiate the identification of devices by approaching a device with a NFC tag of the NFC reader. After identification is over, the user is notified via CAALHP that a new device is available;
- *Locking during installation process*: When a device is being installed, the user shall still be able to use other devices or CAALHP applications, which are not part of installation process;
- *Failure notifications*: The system shall provide a timely response in case of errors and report the failure events. For example, when an application fails to install or integrate with a health device then the end-user and administrator users shall be notified simultaneously. The notification shall also be sent if an application or device fails to respond or crashes during use or execution.

To fulfill these requirements, it is necessary to use a combination of identification and communication technologies that can provide the intended seamless experience. The main purpose of the CRIP is: (1) to identify users and health devices and (2) enhance the use of the latter in a seamless and near zero configuration way. For the first, it must be made on a secure combined device for staff and inhabitant/patient identification; our solution will be based on a combination of RFID and biometric technology (fingerprints). For the second, different types of personal health devices must be able to interface with the CAALHP and the CRIP, using the most recent standards for personal health devices. At the moment there is no truly widely adopted standard for personal health devices. In our perspective, Continua Alliance [39] promises to be a good option since it is based on accepted standards such as IEEE 11073, Bluetooth and USB, and the working group behind it has a strong background in the development of personal health devices. Due to this, our choice was restricted to personal health devices that comply with this standard and whose communication is made via Bluetooth.

As depicted in Figure 1, the CRIP was developed as a separate subsystem deployed on the same AAL space as the CAALHP subsystem. There are two main reasons to develop the CRIP as a separate subsystem: (1) extend the lifespan of the expensive CAALHP subsystem, by extending it in a transparent way to the rest of the system with newer software and hardware technologies; and (2) the necessity to embed dedicated hardware usually not found or with limited access on computers or smartphones. This also allows it to act as an independent gateway for healthcare and medical purposes, meeting the IoT paradigm where small devices can be connected to the Internet through a higher level aggregating device.

As explained, the CareStore platform was designed primarily to be deployed in nursing homes and at patients’ homes. We identified the following actors (human and non-human) that directly interact with CRIP and their interactions with it:

- *Citizen*: A system’s user under care either on a private home or in a nursing home, who can use the homecare platform. A Citizen can seamlessly add a new device by holding it close to the homecare platform and have it install any necessary device drivers and automatically configure them for use;
- *Caregiver*: Can be a doctor, nurse or relative of the Citizen who provides medical treatment to him. Caregivers can add new wireless health devices in a similar way as a Citizen;
- *CAALHP*: Part of the homecare platform that allows integration of different assisted living technologies, making it easier to operate and deploy new AAL and telemedicine devices and applications. CAALHP is connected with CRIP, in order to perform authentication and retrieve data from the personal health devices;
- *Device*: Represents a dedicated personal health device, which is used for monitoring inhabitants in an AAL scenario.

**Figure 2:**
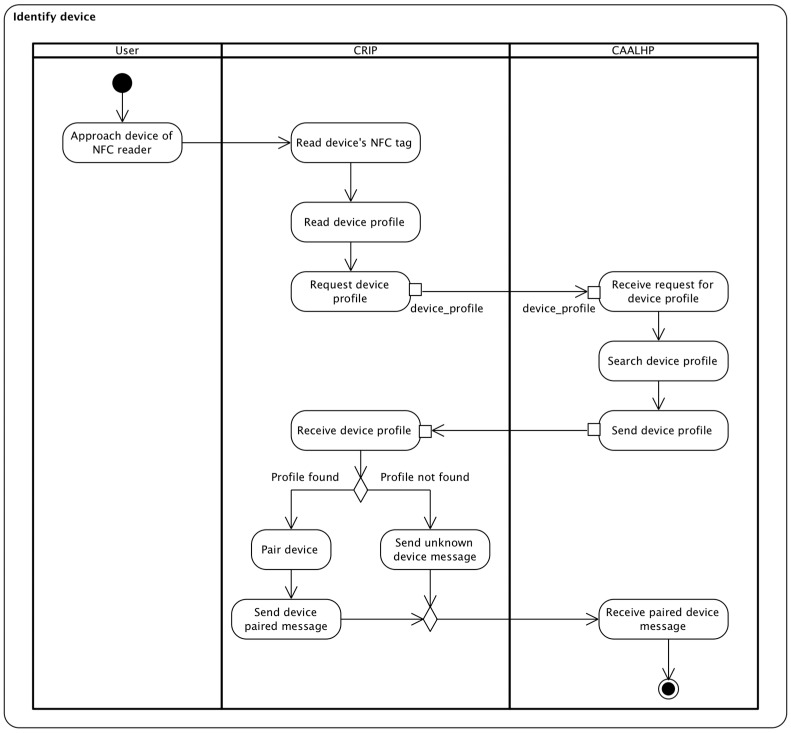
Interaction between the CRIP and CAALHP subsystems of CareStore for identification of a personal health device. The interaction between the Marketplace and CAALHP It is not explicitly depicted, but it is performed when the CAALHP searches for a device profile.

With this set of requirements defined, it is possible to define the set of use cases of the CRIP that need to be implemented. They are listed in Table 2. An example interaction representative of use case UC1 is depicted in Figure 2. The interaction is between the CRIP and CAALHP subsystems when a personal health device is identified. It is important to note that an interaction with the Marketplace also occurs when the CAALHP searches for a device profile, despite not explicitly depicted.

The final important set of requirements for the CRIP relates to the users’ data security and privacy. It was already described that the CRIP will include NFC and biometrics as part of its strategy for identification/authentication of users and devices. A Public Key Infrastructure (PKI) must be defined so that users, devices and external systems should have public and private key pairs to send, receive and validate messages confidentiality and integrity. Some aspects to be considered are:

- The users must register on the platform before they can perform any action on it. For now, this registration process must be made with the presence of another authorized user (e.g., a caregiver). The registration process includes registering the user’s fingerprint and creating an NFC card that can be used for future identification. A combination of fingerprint and card for authentication might only be required for highly secure actions;
- The users’ biometric credentials may be stored locally on the CRIP, for those who are allowed on a particular homecare deployment. This will provide better performance, because the CRIP will be able to authenticate users from the local CAALHP “user profiles” storing authorized users;
- The communications among different subsystems of the CareStore platform must be encrypted and signed to ensure data security. In particular, the health data of citizens is security-sensitive and it is important to secure it during communication.

### 2.2. Hardware Architecture

**Figure 3:**
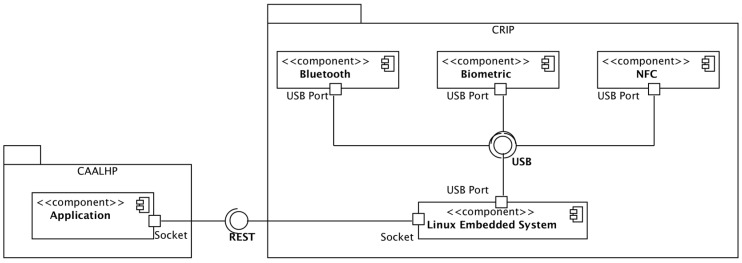
CRIP’s hardware architecture.

Figure 3 depicts the CRIP’s hardware architecture. The integration of the hardware modules is made using an embedded Linux platform that runs dedicated software to handle all interactions and communications between the hardware components and the CAALHP. For external communication with the CRIP, a Representational State Transfer (REST) web service was defined so that the integration of CRIP with other subsystems is faster, as there is no need to develop special device drivers, and it is also flexible enough for future integration with other systems.

Two constraints were defined for the hardware selection: (1) use the maximum amount of COTS hardware and minimize possible modifications, so that the development time is minimized; and (2) choose peripherals with USB connections to ease hardware development and testing. With this in mind, the following hardware peripherals were selected:

- *Bluetooth*: Bluegiga BT111 [40] is a low cost and ultra-small BLE module designed for applications where both Bluetooth Classic and BLE connectivity are needed. The module already includes the USB interface and the antenna. It is capable of detecting devices within a range of 100 m and it is compatible with Linux and the BlueZ Bluetooth stack;
- *Biometric Module*: Suprema Inc.’s SFM3520-OP [41] is an embedded module with a rugged optical fingerprint sensor with a high quality fingerprint image for both dry and wet fingers. It comes with a reliable and fast fingerprint analysis algorithm (matching speed of 1:1 in 800 ms and 1:N in 970 ms) and internal memory that can save up to 9000 fingerprint templates. Although it does not support USB natively, the serial communication can be retrofitted with USB by using appropriate conversion hardware;
- *NFC Module*: The Advanced Card Systems ACR122 [42] is a RFID proximity card that supports the Mifare, ISO 14443 A and B, and FeliCa NFC technologies. It is possible to connect it by USB and the operating distance is up to 5 cm, depending on the type of card and/or proximity tags;
- *Embedded Linux Platform*: Our choice was the Raspberry Pi [43] due to its wide availability and community support. Other platforms were evaluated, namely the Kontron M2M [44] and the Intel Galileo (Gen 1) [45], but they were discarded because of: (1) higher price than the Raspberry Pi, and (2) less support, with the Galileo having, at the time, minimal support due to its early market release, while on the Kontron it was more complex to create and manage Linux images.

### 2.3. Software Architecture

**Figure 4:**
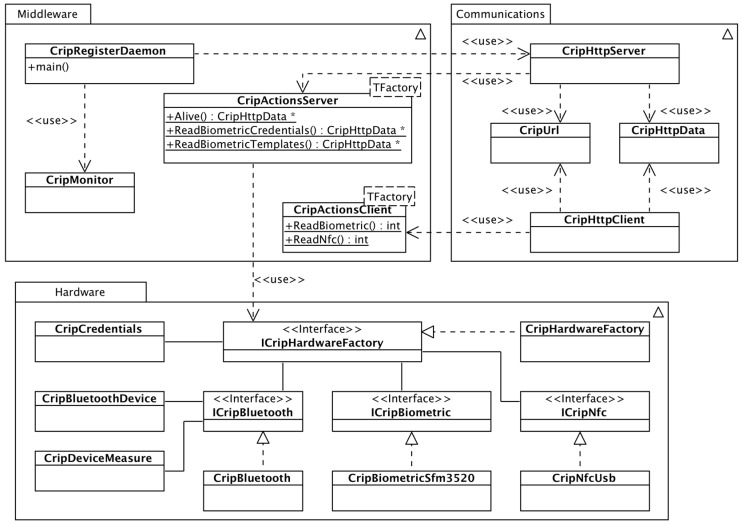
Software architecture for the CRIP daemon.

The software architecture was developed to be flexible so that it supports different versions of the CRIP, i.e., support changes/upgrades to the hardware configuration during the development stages, as well as when installing the platform where different versions could be deployed in order to reduce costs. The chosen operating system was Linux, namely the official Raspberry Pi distribution Raspbian, and the software was written as a Linux daemon in C++ language. The software architecture of the daemon is depicted in Figure 4.

#### 2.3.1. Module Middleware

This module contains all classes that implement the main functionalities of the CRIP. The main class is the CripRegisterDaemon, responsible for initializing all hardware and communications of the CRIP, namely Bluetooth, biometric and NFC, respecting the hardware, and the HTTP server respecting the communications. The initialization of the CRIP is successful if all hardware is available and the HTTP server starts successfully.

When a user interacts with the hardware, an internal interrupt from the respective module (biometric or NFC) is started and the data from the hardware is read. A user interaction can be either of the three: (a) place a finger on the biometric reader; (b) place a smartcard on the NFC reader; or (c) receive an HTTP request from the CAALHP (the user interacted with the CAALHP). All hardware interrupt management is implemented on the class CripMonitor.

CripActionsClient and CripActionsServer are a set of static methods that are invoked when a hardware interrupt or a HTTP request are received. The names refer to where the interruption occurs, respectively, either on the CRIP (client from the HTTP point of view) or remotely on the CAALHP (server from the HTTP point of view). The methods of both classes follow method signatures defined on the classes CripHttpServer and CripMonitor and are passed to both classes upon CRIP’s initialization.

#### 2.3.2. Module Communications

The class CripHttpServer implements a more constrained HTTP server, based on the libmicrohttpd library [46], to handle the REST requests registered when the server starts. When a method is registered it is associated an URL, so that when a REST request is received the respective method is invoked. The class CripHttpClient is a simple HTTP client, based on the libcurl library [47], that allows to perform simple HTTP GET and POST requests to other systems, such as the CAALHP. All data exchanges are made in JavaScript Object Notation (JSON). The class to handle JSON data transformation is also on this module, although not represented on the diagram for brevity and easier understanding.

#### 2.3.3. Module Hardware

The access to hardware peripherals, namely Bluetooth, biometric and NFC reader/writer is abstracted, respectively, by the interfaces ICripBluetooth, ICripBiometric and ICripNfc, and must be accessed through a hardware factory defined by the interface ICripHardwareFactory. The use of interfaces allows implementing and using different hardware, because it abstracts the peripheral’s internal behavior and unifies the access to it with common methods.

The access to the instances of the peripherals is made through the class CripHardwareFactory, an implementation of the ICripHardwareFactory interface. This class abstracts and manages internal configurations of the hardware peripherals, avoiding unnecessary complexity when accessing them. CripBluetooth provides access to the Bluetooth peripherals. It is based on the BlueZ stack [48] and on the Antidote library [49], that handles the reception and parsing of IEEE 11073 messages from Continua Alliance-compliant personal health devices. CripBiometricSfm3520 provides access to the biometric peripheral; the class is based on a custom library where all the actions supported by the peripheral are implemented, since Suprema does not offer official Linux support. The class CripNfcUsb provides access to the NFC peripheral and it is based on the open-source library libnfc [50]. Also on this module, although not represented, are also the classes that permit paring Bluetooth devices using NFC tags. More details on this are presented in Section 2.3.5.

The classes CripCredentials, CripBluetoothDevice and CripDeviceMeasure are data containers for, respectively, handling users’ credentials, Bluetooth information used for configuring the device and handling data readings from the personal health devices. These classes are also used by the class responsible for handling JSON, so the data can be formatted to JSON to be sent to external systems, such as the CAALHP.

#### 2.3.4. Software States Overview

**Figure 5:**
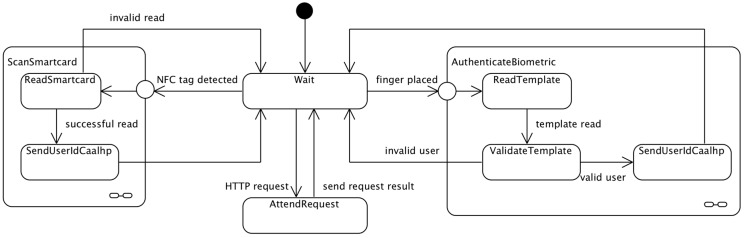
CRIP state machine.

The behavior of CRIP can be described on a simple state machine diagram, as depicted on Figure 5. When the CRIP starts it is always waiting for an interrupt (Wait state), which can either receive a HTTP request or an interrupt from the hardware. When an interrupt is being attended, other incoming requests/interrupts are not attended until the current one is finished. When a HTTP request is received, CRIP attends it (AttendRequest) and then goes back to the Wait state. A hardware interrupt can occur either when a finger is placed on the biometric reader, or when a NFC tag/smartcard is placed over the NFC reader. If the former occurs, the user’s biometric template is read (ReadTemplate), validated against templates previously stored templates on the hardware (ValidateTemplate) and, if the user is valid, its ID is sent to CAALHP (SendUserIdCaalhp); otherwise the template reading is ignored. When a NFC tag is placed on the reader, its data is read (ReadTag) and, if it is successfully read, the information contained on the tag is sent to the CAALHP (SendDataCaalhp); otherwise it is ignored. Both tags with user credentials and devices information are processed accordingly before sending to the CAALHP, so that the HTTP request to CAALHP is called accordingly.

#### 2.3.5. Bluetooth Pairing Using NFC

The Bluetooth standard supports multiple types of pairing, among them the Secure Simple Pairing (SSP) Out-Of-Band (OOB). Although not an official standard, the NFC Forum provides some recommendations on how to implement SSP using NFC [51]; the details of the pairing process will not be replicated here, but we would like to highlight that these are two distinctive and independent processes. The first is a NFC exchange that uses the NFC Data Exchange Format (NDEF) and protocol, to read the device’s information from the NFC tag, and the second is the Bluetooth pairing itself using, in our case, the BlueZ stack. It is important to note that only support for Bluetooth Basic Rate/Enhanced Data Rate (BR/EDR) was implemented, as BLE uses different NDEF payload information for the pairing.

The devices we tested do not natively support any NFC exchange, therefore they were retrofitted with a NFC sticker tag that contained the information need to pair the device, namely the MAC address, pin code and a friendly name for the device. From the point of view of the NDEF protocol, this is considered a static handover, i.e., the communication is unidirectional (the CRIP reads the device’s tag), as opposed to a negotiated handover that can occur when two devices are NFC enabled.

At the time the CRIP was developed a library supporting these data exchanges was not available. Therefore, a custom library was implemented to support them. Details on the library will not be presented here, as it only reflects the data formats defined by the standard. Also new libraries such as the open-source Linux*NFC [52], from Intel, or Open NFC [53], are now available and we recommend their use for future implementations.

### 2.4. Evaluation Protocols

We separate the evaluation protocols into laboratory and on site ones. The last was already presented in Wagner et al. [38]. The laboratory protocols consisted mainly of evaluation protocols of the developed hardware, i.e., laboratory staff interaction with the CRIP and evaluation of personal health devices, according to specified usage scenarios.

#### 2.4.1. CRIP’s Test Application

In both protocols the CRIP tests were made initially with an internal test application, named “Dummy CAALHP”, developed with the purpose of testing individual CRIP functionalities on the absence of a real CAALHP, and with a real CAALHP after its first prototypes were ready. It is noted on the evaluation protocols when each application was used. The application is a simple terminal interface with the following options available:

(1) “Read biometric templates” allows reading user fingerprint templates from the biometric hardware, so they can later be stored. For each user, two biometric templates are read to improve fingerprint recognition due to finger placement;
(2) “Store templates” allows storing user’s templates collected in option 1. The templates are stored on the biometric hardware and a user is recognized once he tries to authenticate at any moment;
(3) “Scan for Bluetooth devices” performs a scan for Bluetooth devices near CRIP. This option is used mainly for development and test purposes;
(4) “Write UID on NFC card” writes the user’s UID data on a NFC tag;
(5) “Write Bluetooth data on NFC tag” writes the Bluetooth device’s information on a NFC tag that can later be used to identify and pair a Bluetooth device;
(6) “Write device data on NFC tag” writes custom information regarding a non-Bluetooth device on a NFC tag, so that the device can be identified by the CRIP;
(7) “Check CRIP status” sends an alive message to CRIP to check its current status.

When a NFC tag of a device is placed on the CRIP and the corresponding device is paired, that information is sent to Dummy CAALHP and a new option is available. That option enables to request the CRIP to acquire data from the identified health device.

At any moment, the user can perform an authentication either by placing a finger on the biometric reader, or by placing a tag on the NFC reader. When a smartcard is valid or a biometric credential is recognized, a message is received by the applications’ HTTP server running in background and it is presented on the screen. The exchanged messages are presented on screen on JSON format.

#### 2.4.2. Protocol for Users’ Identification

The CRIP was used as identification platform on the UMinho’s laboratory during its development stage. This situation simulated a scenario of a nursing home were multiple users register in the CareStore platform and can access a CAALHP installed on a citizen’s room. Both CRIP prototypes were used for this evaluation.

**Figure 6:**
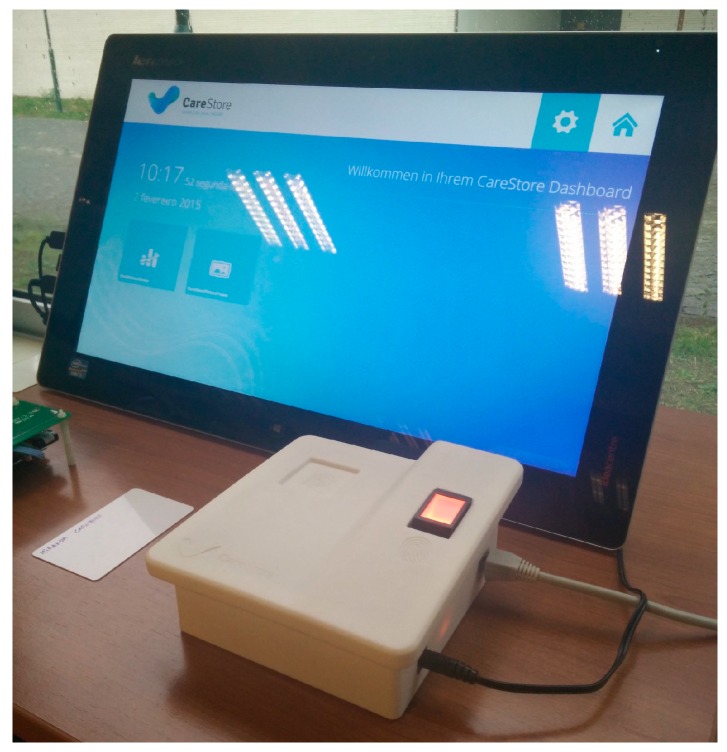
CAALHP and CRIP setup used for the evaluations.

Permission was requested to register all the laboratory’s staff on the platform; thus 15 subjects with an average age of 23 years, all of them acquainted with the use of technology were registered. The registration of each member was made with the help of the facilitator of the evaluation, using the Dummy CAALHP application. It was requested to record a fingerprint of its choice, storing it as a template only on the biometric hardware, and a NFC card with a random Unique Identifier (UID) was also recorded and handed to the person. Then, it was requested from the subjects that when arriving to the laboratory in the morning and when leaving it in the evening, they authenticate themselves on the platform using both the NFC and fingerprint mechanisms. The feedback that a user was authenticated was given on the CAALHP application. The test setup comprised of a CAALHP and a second generation CRIP prototype is depicted in Figure 6.

This evaluation lasted for about eight months to the end of the project in February 2015. Qualitative verbal evaluations were performed during the duration of the evaluation, in order to understand how the users felt about using the platform, and specifically the interaction with the CRIP, and if some issues were occurring with it.

#### 2.4.3. Protocol for Devices Identification and Communication

As stated, the CareStore platform is able to communicate seamlessly with personal health devices that support the Continua Alliance standards. Two devices were selected for this evaluation: the A&D UA-767PBT-C [54], a blood pressure monitor, and the A&D UC-321PBT-C [55], a weight scale. The UA-767PBT-C is a fully automatic digital blood pressure monitor that supports Bluetooth 2.1 and it is capable to save up to 25 measurements. The UC-321PBT-C has a 200 kg capacity with a resolution of 100 g. Extended functionality is provided by Bluetooth version 2.1 wireless communications.

The first two evaluations were performed by two of the authors at the University of Minho’s laboratory, during the development stage of the CRIP. Both devices were retrofitted with a NFC tag containing pairing information described in Section 2.3.5. The evaluations were performed sparsely between September 2014 and February 2015.

The objective of the first evaluation is to simulate a nursing home, where a nurse responsible for multiple citizens would carry health devices that would be used to acquire vital signs from the citizens. Therefore, two setups similar to the one depicted in Figure 6 were used, each simulating a different room, with the CAALHP running the Dummy CAALHP application. Initially the devices were unpaired. When a nurse arrives to a room, the first step was to approximate the tag to the CRIP in order to identify the device and pair both, if that was not the case. This process must be repeated for each of the devices, in this case the blood pressure monitor and the weigh scale. After that, the nurse would acquire some vital data readings, in this case blood pressure and weight. When this process is finished, the nurse would go to the next room, where the same process of identifying a device and acquiring vital data is repeated. This entire process would be similar for a citizen’s home deployment with a visiting nurse taking its devices to acquire health measurements.

A second evaluation reviewed a home deployment scenario, where a citizen has access to a CAALHP connected to a CRIP and some health devices, and it is responsible for its health measurements. A deployment such as the one presented on Figure 6 was used for the evaluation, with the CAALHP executing Dummy CAALHP. The NFC tag that identifies a device was placed on the CRIP in order to identify it and if necessary also pair it. After they were paired, the Dummy CAALHP application was used to acquire vital data from each device. This identification and data acquisition procedure was repeated five times for each device.

A third evaluation to assess the systems seamless device configuration was performed in the Aarhus University laboratory by three of the authors, using a custom health device, namely an intelligent bed. Specifically, the CRIP’s device identification functionality using NFC tags and the device profile installation and configuration on the CAALHP are evaluated. A setup similar to the one depicted in Figure 6 was used, with the CAALHP running both CAALHP and Dummy CAALHP applications. First a NFC card was written with the device’s information, using the Dummy CAALHP application. After, the user must authenticate on the system, via biometrics, so it unlocks and allows the installation of new devices. On successful authentication, the NFC tag containing the bed’s profile was placed on the CRIP and the profile information is sent to the CAALHP. Last, this must connect to the CareStore Marketplace and download, install and configure the respective device driver and application. On this protocol, the process must fail if an unauthorized user tries to install the device.

## 3. Results

### 3.1. CRIP Prototypes

Two CRIP prototypes were developed during the project. The first prototype was a quick assembly to mainly test integration with the CAALHP, while the second prototype resolved some issues that appeared during the first one and received some hardware upgrades.

**Figure 7:**
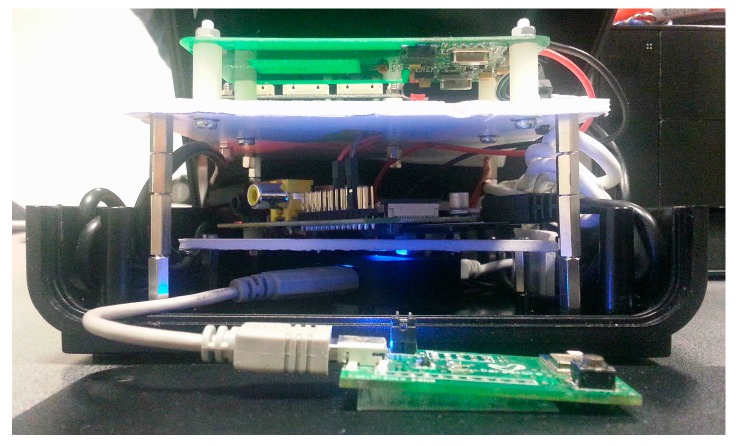
Hardware configuration of the first prototype.

**Figure 8:**
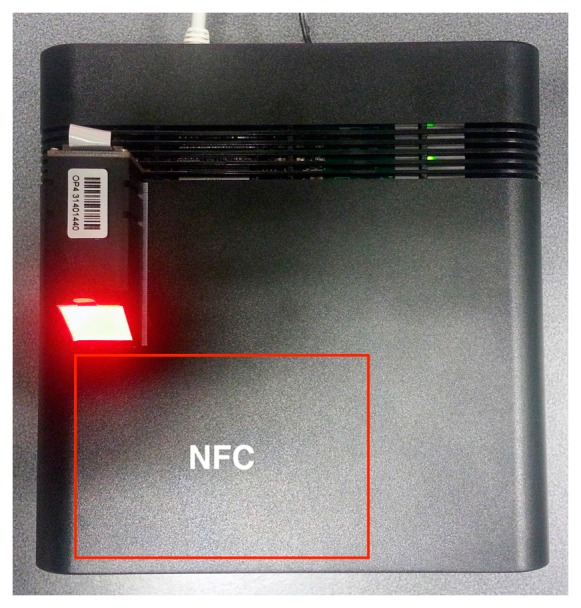
Top view of the first prototype.

In the first prototype the hardware was distributed in three levels, as depicted in Figure 7. The first level is solely an USB hub, the second one a Raspberry Pi Model B and the third one is composed of the NFC and biometric modules; the Bluetooth module was attached to the front panel of the box. The NFC and Bluetooth hardware modules where connected to the USB hub, while this and the biometric module where connected to the Raspberry Pi’s USB ports. On this prototype a USB hub was used because this Raspberry Pi model only had two USB ports available to connect all hardware peripherals. Some problems with the recognition of USB peripherals occurred on the Linux, when all of them where connected to the hub. These issues were probably related with powering the peripherals, therefore the option to connect just the Bluetooth and the NFC to the hub, which did not present any issues. The Ethernet connection is extended to the back panel of the box. The top view of the final assembly is depicted in Figure 8. The red area symbolizes the recommended place for the NFC tags to be placed and the biometric fingerprint reader was placed on the left of the device. Although not ideal, this option was taken due to lack of internal space on the box and because there was need to had a support to hold it.

**Figure 9:**
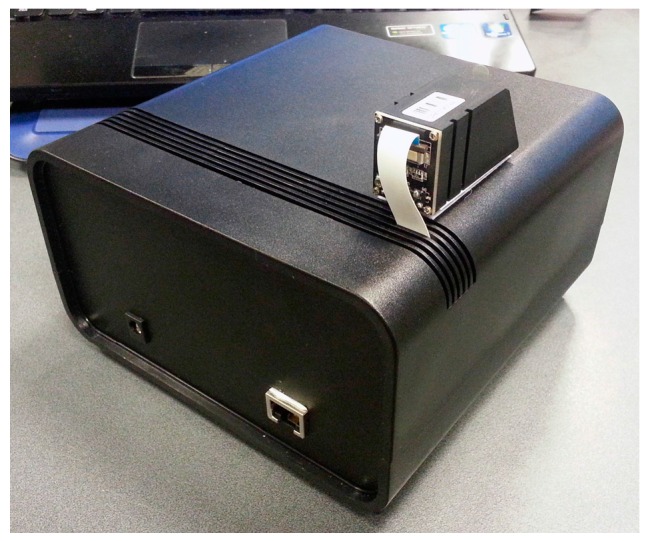
Rear view of the first prototype.

**Figure 10:**
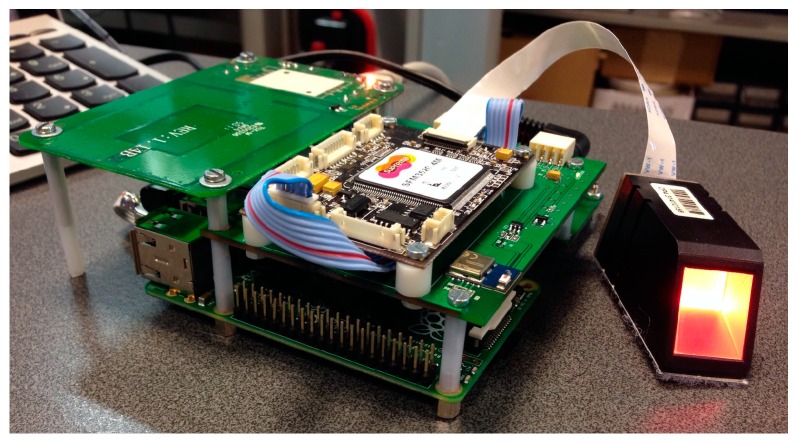
Hardware configuration of the second prototype.

**Figure 11:**
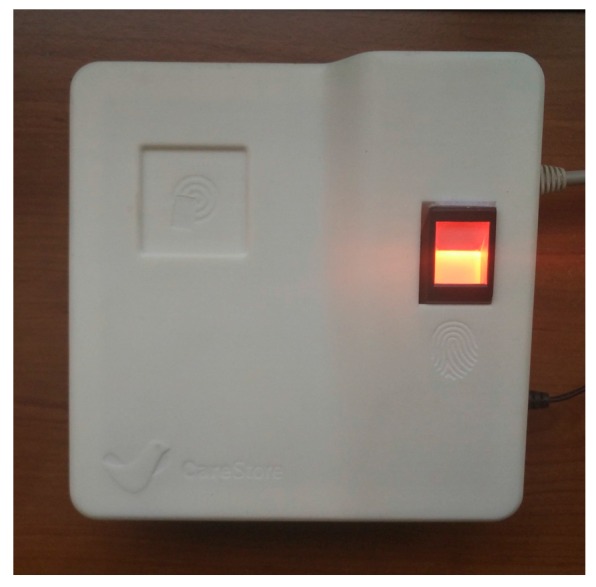
Top view of the second prototype.

**Figure 12:**
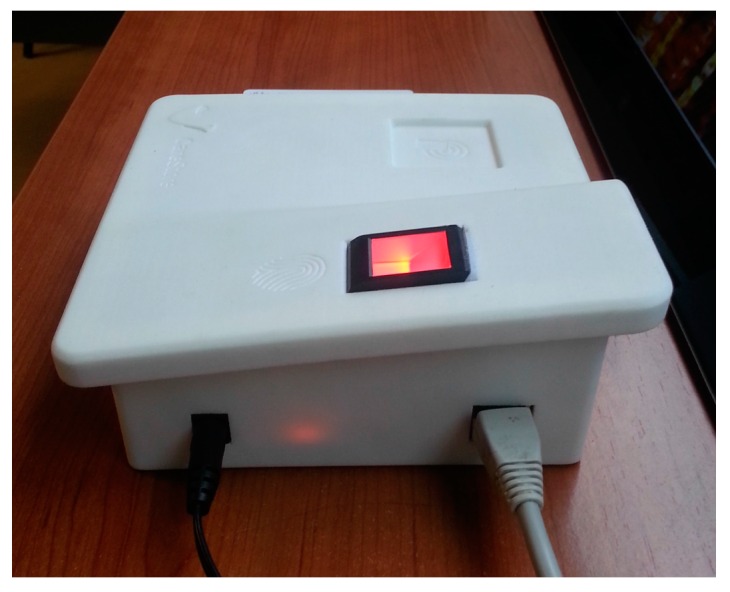
Side view of the second prototype.

Figure 9 shows a rear view of the first assembly with the connectors for the power supply and the Ethernet communications. In the second CRIP prototype the major modification was the hardware configuration that was made to be installed on a custom designed box to be deployed on the test site. This configuration is depicted in Figure 10. The top and side views of the box are depicted in Figure 11 and Figure 12, respectively. The modifications made on the second prototype are:

- A Raspberry Pi Model B+ was used, instead of the previous Model B. This model started to be commercialized during the project’s development and was adopted because the Model B would be discontinued. Also, the dimensions and connections of the board allow a more compact design;
- The Bluetooth module is now integrated on the same board as the biometric module. This option was chosen to reduce the hardware size;
- A new custom power and USB hub board was developed. The power part provides 5 V from the main power supply to all hardware peripherals of the system, enhancing the voltage and current stabilization on all boards. The USB hub circuit is similar to the Conceptronic USB hub used on the first prototype. Our option to keep using it was mainly based on the need to eliminate possible powering issues of the first prototype, which was successfully done. All peripherals were connected to the hub and this was connected to the Raspberry Pi through USB;
- The operating system was stored on a USB pen drive, while on the first prototype was on a SD card. This option was made due to the flash memory architecture used by a pen drive, which offers better resilience against file system corruption in case of failure such as a power outage. Still, a SD card was needed for booting the Raspberry Pi’s Linux.

### 3.2. Users Identification and Authentication

All subjects complied with the described protocol during the evaluation period in the laboratory. Once the protocol was described to each member of the staff team, they did not report any problems with it. When asked if they felt that the authentication process was easy and if they did not have any difficulties complying with it, all participants agreed. Some issues regarding CRIP inoperability were sporadically reported, which most of the times occurred due to software bugs that were corrected. When asked if they could understand the applicability and advantages of the system, all users answered positively and could imagine using it in the future for managing their own health.

### 3.3. Health Devices Identification and Communication

After the NFC reading software was stabilized, the identification of the personal health devices using NFC tags worked every time the device’s identifying tag was placed on the CRIP, for all three evaluations. The first evaluation presented some issues as the pairing process did not work as expected for the designed protocol. On the first pairing try, i.e., the device has not been previously paired with a CRIP or any other device, the process worked without any issue. When trying to pair the same device with a second CRIP, the process failed, although the pairing information was correctly read from the NFC tag. This pairing process failed for both tested devices. We believe this was due to their design. This topic is discussed further in Section 4 of this article. For the second evaluation, the devices were always recognized and it was possible to acquire health data from them.

The identification and configuration of the custom device was successful, i.e., the CAALHP received the device’s profile information from the CRIP and was able to download, install and configure the device driver and application of the intelligent bed on the CAALHP, without any intervention by the facilitators. The protocol was tested for both authorized and unauthorized users and worked as expected, i.e., authorized users could install the device’s software, while unauthorized ones failed to do so.

## 4. Discussion

The two presented prototypes served to demonstrate that secure and seamless interaction with health devices and services is feasible, by using standards and IoT enabling technologies, in this case NFC, biometrics (fingerprints) and Bluetooth. NFC is key on the CRIP, because it is the main automation enabler for near-zero device configuration and interaction, an important aspect of the IoT paradigm [18,21,22]. Compared with other presented works [29,30,31,32,33,34], this distinguishes itself by focusing on how the merge of identification and communication technologies on a gateway can drive the seamlessness of the whole platform, while the referred works focus mainly on the integration, communication and use of sensors on healthcare systems. Also, the CareStore project adopted the concept of “store”, now a common solution born and widespread on the mobile area, and tried to adapt it to the needs of the healthcare area and assess its benefits and advantages. Such integration can enhance users experience and therefore, we believe, make them adopt health technology on their daily routines, which ultimately may enhance their well-being. On a medical perspective the major difficulty for such platforms is inherent to the specific nature of each medical condition that requires different forms of action. In the end, these difficulties are reflected on the technological solutions that make a one-size fits all one very difficult to achieve. Our evaluations on the combination of NFC, biometrics and Bluetooth, three of the most promising IoT enabling technologies provided optimistic results. The evaluations in laboratory and with the caregivers on the nursing home increased our confidence in these, as the users generally agreed that: (1) the identification/authentication process was easier and less distracting; and (2) interfacing with personal health devices was easier. The participants also generally agreed that they could imagine using similar platforms in the future for their own health management and that they could increase their acceptance and adherence to their daily health routines, ultimately improving their well-being.

As a blueprint platform for projects on the healthcare IoT area, we believe the CRIP to be a disruptive one due to its compliance with the two main characteristics presented on the IoT definition by Xu et al. [18]: Self-configuration and standard and interoperable communication protocols. This makes it an interesting platform for research, as it can be used in different environments and support studies on health management for various medical conditions. When designing it, we tried to create it as an agnostic platform for identification and communication, that can be used to interface with several types of sensors. Since the intelligence of the system is mainly supported by applications installed on the CAALHP, the platform can enable management of very different medical conditions. It can also be extended and adapted to other healthcare platforms with the addition of newer sensor and communication interfaces, a step to promote the IoT’s systems scalability and seamless integration of “things” onto them. We consider this the major contribution of our work since we could demonstrate that its integration is feasible and can bring benefits to the healthcare workflows. It is also worthy of note that such platforms, in the future, do not necessarily need to be a device such as the presented one, but they can be embedded into different systems such as personal computers, smartphones, televisions, set-top boxes, cars, furniture, etc, enabling the pervasiveness of healthcare IoT systems.

Some of the IoT aspects that were met by the CRIP that we would like to highlight are availability, scalability, privacy and security. Regarding the availability and scalability of IoT solutions, with the growth of personal health devices, boosted especially by the number of fitness and health wearables, it is important to support them in a seamless way in order to enhance the technological user experience and well-being. We believe that that comes from platform independency and continuously available services and applications that can involve citizens on health related routines. While this is difficult to achieve, it can be easier, from the technological point of view, if the device manufacturers can be involved on this integration. As part of the CareStore strategy, that could be possible through the development of medical applications for the CAALHP and creation of device drivers that support communication and data handling with the devices. Regarding the security and privacy there are two points to highlight: (1) the security of users can be enhanced with the combination of NFC and biometrics, with the first providing a lower-level identification, while the second restricts the system to higher-level authorized users; and (2) the users’ privacy was ensured locally by not storing any sensitive information—personal data such as names, addresses or IDs, as well as vital records—that could be related to a user. This topic is further discussed in Section 4.1 as part of the limitations of the system.

Another aspect that distinguishes our solution from others is its openness and standard compliance, since it is based on open and/or relatively affordable hardware, which makes it easy to build by other teams interested in explore this type of platforms for IoT in healthcare. As presented in previous research [18,21,22,56], the IoT concept can best proliferate if more standards and open solutions are adopted. This is a position with which we strongly agree, therefore our choice of using open hardware, open software and major standards. On the hardware costs of the CRIP, they were relatively high, but mainly due to the cost of the biometric module, which represented about 50% of the total cost. The remaining hardware could be easily acquired and assembled. Other options were considered for the different hardware peripherals, but the constraints to USB devices to ease the assembly while making it flexible, limited our options to the ones presented. It is interesting to note that the last Raspberry Pi 3 already includes Bluetooth and Wi-Fi, which can make the solution even cheaper and simpler to develop.

### 4.1. Limitations

These prototypes still present various limitations from the architectural and technical point of view, as well as from the evaluation one. We will start by point out the technical ones, followed by the limitations of the evaluations.

The first limitation is the direct connection via Ethernet of the CRIP with the CAALHP, with the first only be able to communicate with the second. It was the authors option to do it so on these first prototypes, so that the development of the platform was easier and better focused on developing the functionalities of the CRIP and the CAALHP. Introducing Wi-Fi for communications would imply further installation of equipment, increasing the costs of the final solution and deployment, and also demand integration of discovery protocols implying extra efforts in securing the communications and especially on identifying and authenticating genuine CRIPs towards CAALHPs. By using Ethernet communications, we ease the deployment and identification, as the CRIP and the CAALHP are on the same space. Still we cannot reliably assure that a genuine CRIP is connected to a CAALHP, as someone can connect an unauthorized device, or perform a man-in-the-middle type of attack. Despite this, we believe that wireless technologies can provide a more seamless experience and as future work we intend to integrate Wi-Fi and the necessary mechanisms for communications and security between systems.

The second limitation relates to the type of personal health devices evaluated. The option to only use Bluetooth and Continua Alliance-compliant devices did not raise major issues when interacting with them, especially due to the Antidote library. Nevertheless, we found some limitations regarding pairing them according to the protocol described on Section 2.4.3. We were able to identify and pair a device with the CRIP if it was unpaired, but a second pairing attempt with another CRIP failed. We did not delve much into this issue, but we could assess that if a device was unpaired after the first paring, we were able to pair it again. We think this is a limitation of the evaluated health devices that were only able to pair themselves with only one device. The unpairing process for the evaluated health devices required to remove the batteries (we presume to erase the device’s memory) and repeat the pairing process. We consider this a big limitation for the intended system operation, as this pairing/unpairing process is distracting and cumbersome for a nurse or a citizen that may: (1) not be acquainted with these technological processes and (2) want to have a seamless and effortless experience with the technology. We noticed that the same manufacturer already offers new devices that, they state, can be paired with multiple devices, but it is an open question that still needs further evaluation.

As a third limitation we would like to point out some issues regarding the security and privacy of the CRIP, as they can be helpful for designers of similar platforms, although we will not delve much in these topics. On these prototypes, three security mechanisms were used, namely fingerprints for users’ authentication and identification, NFC for users and devices identification, and HTTPS for communications between the CRIP and the CAALHP. Although these mechanisms provide a good level of security on the system, they are not enough, especially from the privacy point of view. On the users’ identification/authentication, the fingerprint can be considered one of the best that exists and the selected module provides a good level of security since the fingerprints can be stored on hardware, which makes its cracking more difficult, but fingerprint distribution still raises many questions as they can pose major security failures due to unauthorized access and compromise users security and privacy. This is a topic that still needs further research. On the use of NFC, we can only guarantee identification, not authentication [57]. This is an inherent problem of the technology that, in our opinion, can only be managed by setting a proper PKI. During the development, we did not address data exchanges security on read and write NFC tags, despite their availability. This is something that we intend to address in future developments of the CRIP. Finally, a word on the users data privacy. At this point, the CRIP had implemented a minimal privacy mechanism. The only data stored on a local database are the user IDs from the NFC cards, associated with a random fingerprint ID that was generated when a user enroll on the system. It is important to note that the fingerprint templates were always stored on hardware and never on any other support. Hardware security mechanisms can also be added to further improve the CRIP security against tampering, specifically for handling certificates and store sensitive information [58]. These can be used in combination with the presented identification mechanisms to increase security and privacy, i.e., the identification mechanisms can provide unique encryption salt values, especially the biometrics, that are associated with a unique user, that can be used to encrypt and decrypt stored user information [59]. In case of a remote attack, accessing user’s data is still possible, but because the data is encrypted it becomes unusable. Still, these strategies and mechanisms on themselves do not make the system bulletproof and more thorough research needs to be made on these topics.

The fourth, and last, limitation relates to the evaluations. Although it is our intention that this platform can be used both in nursing homes as well as at a patient’s home, we only evaluated it in the first scenario. The major factor to do so is that the environment is more controlled, when compared to a household, as nurses are available on a known environment and the citizens’ routines are also known and well established. This limits any conclusions of the project to these environments, as healthcare management on a patient’s household or on healthcare facilities such as hospitals can be very different. In a household the healthcare management and devices interaction will be performed mainly by the inhabitants, while the second one different healthcare personnel can intervene, such as doctors, nurses or caregivers, all of them with different requirements. Also, the health devices can be completely different in both cases. In a household scenario scattered readings from small devices such as handheld blood pressure monitors, glucose meters or wearables, may be the main data providers; in healthcare facilities devices for continuous monitoring such as EMG, ventilators or pulse oximeters, might be used to monitor the patient. With this in mind, we suggest care when using the results of the presented work.

## 5. Conclusions

An innovative design of a gateway named CRIP for the healthcare IoT was presented. Two prototypes were designed, implemented and evaluated. The evaluation results were presented to illustrate the functionality of the platform, how it can be integrated on the IoT and how users understand it. On this last point, the participants of the evaluations understood the purpose of the gateway and could image using a similar device for their own health management. The major contribution of this work when compared with previous ones, is the demonstrated feasibility of integrating standard and structural IoT identification and communication technologies that enable near-zero configuration and communication standardization of “things” on the healthcare domain. Another contribution of this work is that is addresses the presented challenges, namely health device integration, security and privacy, that other researches may find while developing their platforms. As future work we intend to keep integrating new ICTs on the CRIP, as its support makes the device more interesting for rapid prototyping and evaluation of sensor technologies on healthcare case studies, also because research on e-Health areas will have a focus on the evaluation and application of these technologies on the healthcare scenarios.

## Figures and Tables

**Table 1:** Resume of the deployment scenarios and technologies used of the presented projects.

Platform	Deployment	Communications	Identification	Standards	Cloud
Rahmani et al. [30]	Hospital, Home	Bluetooth, Wi-Fi, 802.15.4/6LoWPAN	-	IEEE 1073	Yes
Catarinucci et al. [31]	Hospital	802.15.4/6LoWPAN	UHF-RFID	-	No
Cubo et al. [32]	Hospital, Home	802.15.4	NFC	-	Yes
Saponara et al. [33]	Nursing home, Home, Pharmacy	Bluetooth/BLE, Wi-Fi, 3G	-	HL7 CDA	Yes
Yang et al. [34]	Home	Wi-Fi, ZigBee	UHF RFID	-	Yes
Ghose et al. [35]	Home	Bluetooth, Wi-Fi, 3G	-	-	Yes
CRIP	Nursing home, Home	Bluetooth, Ethernet	Biometrics, NFC	Continua Alliance	Yes

**Table 2:** List of use cases of the CRIP.

ID	Description
UC1	Health devices shall be seamlessly detected by the CRIP.
UC2	Authenticated users shall be able to logon to the CAALHP via the CRIP.
UC3	Health devices shall be defined by a Device Profile that is related to a CareStoreDeviceDriver in the CareStore Marketplace.
UC4	CAALHP may communicate with health devices via the CRIP.
UC5	Vital signs from health devices may be collected via the CRIP or the CAALHP.
UC6	Read users’ credentials in order to be stored on the CareStore Marketplace.
